# Clinical outcomes of bortezomib-based therapy in myeloma

**DOI:** 10.1371/journal.pone.0208920

**Published:** 2018-12-12

**Authors:** Faouzi Djebbari, Anandagopal Srinivasan, Grant Vallance, Sally Moore, Jaimal Kothari, Karthik Ramasamy

**Affiliations:** 1 Department of Clinical Haematology, Oxford University Hospitals NHS Foundation Trust, Oxford, United Kingdom; 2 NIHR BRC Blood Theme, Oxford, United Kingdom; 3 Oxford Myeloma Centre for Translational Research, Oxford, United Kingdom; PLOS, UNITED KINGDOM

## Abstract

Bortezomib, a first generation proteasome inhibitor, is used in both newly diagnosed and relapsed myeloma settings. Considerable differences exist in the usage of bortezomib therapy in the clinical practice setting in comparison to clinical trial setting as well manufacturer’s recommendations. These differences include route of administration (intravenous (iv) vs. subcutaneous (sc)), frequency from twice to once weekly, choice of alkylating agent used in combination with bortezomib (melphalan or cyclophosphamide), and choice of glucocorticoids (dexamethasone or prednisolone). We reviewed data from 272 consecutive bortezomib-treated myeloma patients, who received therapy within the regional Thames Valley Cancer Network for both newly diagnosed myeloma (NDMM, n = 120) and relapsed MM (RMM, n = 152). We investigated the influence of age, sex, transplant, bortezomib combinations (doublet vs. triplet), cumulative bortezomib dose per treatment line (<50mg vs. ≥50mg), and route of administration (iv vs. sc) on time to next treatment (TTNT) and on overall survival (OS). Route of bortezomib administration (iv vs. sc) influenced neither OS (41 vs 35 months, p = 0.5), nor TTNT (14 vs. 19 months, p = 0.052). Our study showed a statistically significant improvement in median OS in patients receiving a cumulative dose ≥50mg compared to <50mg (42 vs. 33months, p = 0.003), although presence of confounders need to be taken into account, such as disease stage, performance status, genetic changes and prior therapies. Median OS was longer using triplet therapies compared to a doublet in the RMM cohort (37 vs. 29 months, p = 0.06), although this did not reach statistical significance. Multivariate Cox Regression analysis showed that cumulative bortezomib dose ≥50mg (p = 0.002, HR = 1.83, 95% CI 1.25–2.67) and autologous transplant (p = 0.002, HR = 2.6, 95% CI 1.41–3.98) were both significant factors associated with improved OS. Our data argues in favour of continuing bortezomib for the recommended duration as per Summary of Product Characteristics (SPC), subject to good tolerability, in order to deepen response or extend the duration of best response.

## Introduction

Multiple myeloma (MM) is a haematological malignancy characterised by uncontrolled proliferative behaviour of clonal plasma cells [1, 2]. It remains an incurable disease with a relapsing and remitting course [3]. However, it has become increasingly more treatable with resultant improvement in survival. The last 10 years, witnessed approval of new anti-myeloma therapies from distinct pharmacological classes, such as immunomodulatory drugs iMiDs (e.g. lenalidomide, pomalidomide), proteasome inhibitors (e.g. bortezomib, ixazomib), histone-deacetylase inhibitors (e.g. panobinostat) and monoclonal antibodies (e.g. daratumumab).

Bortezomib, the first-in-class proteasome inhibitor, remains a widely used anti-myeloma agent, despite licensing of newer agents. The use of bortezomib in combination with other agents, is standard of care and is approved by the UK’s National Institute for Health and Care Excellence (NICE) to treat NDMM, in the transplant eligible (TE) setting (at 1.3mg/m^2^ (days 1, 4, 8 and 11 of a 21 days cycle for up to 24 doses over 12 weeks) in combination with dexamethasone as per IFM trial; or at 1.3mg/m^2^ (days 1, 4, 8, and 11 of a 21 days cycle for up to 24 doses over 24 weeks) with dexamethasone and thalidomide, as per PETHEMA trial) [4]. Bortezomib is also NICE-approved to treat NDMM in the transplant non-eligible (TNE) setting (at 1.3mg/m2 (days 1, 4, 8, 11, 22, 25, and 32 of a 35 days cycle during cycles 1–4, and days 1, 8, 15, 22 during cycles 5–9, up to 12 treatment cycles for a total of 52 weeks); in combination with an alkylating agent (melphalan) and a corticosteroid (prednisolone), as per VISTA trial)[5]. In the relapsed MM setting, bortezomib is NICE-approved (at 1.3mg/m^2^ (days 1, 4, 8 and 11 of a 21 day cycle, for up to 8 cycles or a maximum of 32 doses over 24 weeks) in combination with dexamethasone as per APEX trial)[6].

A phase III multicentre study randomised 222 relapsed myeloma patients to either iv or sc bortezomib given twice weekly (days 1, 4, 8 and 11 of a 21 days cycle) at 1.3mg/m2 per dose and for up to 8 cycles [7]. The study showed that bortezomib sc offers non-inferior efficacy to iv route [7]. However, peripheral neuropathy (PN) was significantly less common in the sc arm compared to iv arm: PN of any grade (G) was (38% vs. 53%, p = 0.044), G2≥ (24% vs. 41%, p = 0.012), G3≥ (6% vs. 16%, p = 0.026) [7]. Lower peak serum concentration (Cmax) of bortezomib from subcutaneous administration partly explains the lower neuropathy rates [7].

A post-hoc analysis of a phase III bortezomib trial investigated safety and efficacy of bortezomib given once weekly compared to twice weekly [8]. Long term efficacy outcomes appeared to be similar. However, the incidence The incidence of G 3/4 PN was significantly lower in the weekly bortezomib arm (8% vs. 28%, p< .001) [8].

A phase II trial evaluated three low-dose intensity sc bortezomib-based treatments in patients ≥75 years with NDMM. Patients received sc bortezomib plus oral prednisone (VP) or VP plus cyclophosphamide (VCP) or VP plus melphalan (VMP), followed by bortezomib maintenance, and half of the patients were frail [9]. This study demonstrated that VP, VCP and VMP regimens demonstrated no substantial difference in efficacy. However, toxicity was higher with the melphan-containing regimen (VMP), particularly cytopenias [9].

Based on the above data a number of key changes have been implemented in clinical practice to improve tolerability and optimise outcomes of bortezomib-based therapies (e.g. dosing frequency from twice weekly to once weekly dosing, choice of alkylating agent either melphalan or cyclophosphamide, and the switch from intravenous to subcutaneous administration to reduce peripheral neuropathy).

In addition, triplet therapies, which consist typically of bortezomib plus a steroid in addition to either an alkylating agent or an IMiD, are favoured over doublets (bortezomib with dexamethasone); in line with improved efficacy demonstrated in phase 2 studies [1, 10]. NICE approvals in both myeloma settings are backed by efficacy evidence from large phase III studies. In TNE NDMM setting, for instance, VISTA trial showed that bortezomib plus melphalan and prednisolone (bortezomib group) was superior to melphalan and prednisolone alone (control group) in TNE NDMM patients; median time to progression (26 vs. 16.6 months, P<0.001) [11]. The proportions of patients with a partial response or better were 71% in the bortezomib group and 35% in the control group [11]. Complete-response rates were 30% and 4%, respectively (P<0.001) [11]. In RMM setting, Apex trial demonstrated that, bortezomib plus high dose dexamethasone was superior to high dose dexamethasone alone with an overall response rate (38% vs. 18% (P<0.001)) and median time to progression (6.22 vs. 3.49 months (HR: 0.55; P<0.001)) [12].

However, in clinical practice, a number of bortezomib-based regimen use different schedules to those in the previously mentioned large phase III studies (change of frequency, different choice of alkylating agent or corticosteroids). Their use in clinical practice can be considered off-label but their efficacy and tolerability was demonstrated in clinical studies [7, 8, 9]. Examples include bortezomib at 1.3mg/m^2^ (days 1, 8, and 15) in combination with cyclophosphamide and dexamethasone (VCD) [1], bortezomib at 1.3mg/m^2^ (days 1, 8, 15, and 22) in combination with bendamustine and dexamethasone (BVD) [1], and bortezomib at 1.3mg/m^2^ (days 1, 8, 15, and 22) in combination with dexamethasone [1].

Often clinical outcomes observed in clinical practice are inferior to what has been reported in clinical trials. As usage of bortezomib in clinical practice varies with SPC guidance, we set out to audit clinical outcomes of bortezomib usage in clinical practice both in NDMM and RMM setting in a large cohort of patients treated within the regional Thames Valley Cancer Network (TVCN) in the UK.

## Materials and methods

### Study design and inclusion criteria

We reviewed records of 272 consecutive MM patients treated with bortezomib-based regimen between 2010 and 2016, either at NDMM or RMM setting. Inclusion criteria were adult patients with a diagnosis of MM, treated within TVCN with bortezomib-based therapy either at first line or at relapse.

### Data collection

Clinical data was collected from the chemotherapy prescribing database and patient records. The following patient demographic data were collected: age at start of bortezomib therapy (<75 years, ≥ 75 years), sex, and autologous stem cell transplant (ASCT) status. The following treatment characteristics were collected: bortezomib regimen, treatment combination (doublet, triplet), cumulative bortezomib dose received per line of therapy (<50mg, ≥50mg) and route of administration (intravenous, subcutaneous). Detailed toxicity assessment data was not available, but the proportion of patients discontinuing therapy due to toxicity was recorded.

### Outcome measures

Two clinical outcomes were measured: overall survival (OS) and time to next treatment (TTNT), both of which are justifiable endpoints for this cohort of patients. OS is important because myeloma is an incurable disease, and prolonging survival is a key treatment goal. In addition, treatment of elderly myeloma patients who present with co-morbidities can cause significant toxicities that often limit survival. TTNT is of paramount importance for myeloma patients because time off-therapy in remission (partial or complete) is often associated with improved quality of life and less visits to the hospital. OS was calculated in months from start of bortezomib therapy until censor date or death. TTNT was calculated in months from start of bortezomib therapy until start of subsequent line of therapy or censor date, whichever is earliest. This study investigated the impact of the following factors on OS and TTNT: sex, age at start of therapy (<75 years vs. ≥ 75 years), ASCT status, choice of combination regimen (doublet vs. triplet), cumulative bortezomib dose (<50mg vs. ≥50mg), and route of bortezomib administration (sc vs. iv).

### Statistical analysis

OS and TTNT were presented in KM curves with medians (in months) and p values. Univariate analyses using log-rank test and Cox regression method were performed for total cohort, NDMM and RMM cohorts to assess the impact of the following factors on OS: sex, route of administration, age, combination, transplant and cumulative dose. Multivariate analysis was also performed to assess the impact of the following factors on OS: sex, line of therapy, age, combination, transplant and cumulative dose.

## Results

Patient characteristics (Table 1) were comparable between NDMM and RMM cohorts in terms of sex, transplant status, route of bortezomib administration, and cumulative dose. There was a higher proportion of patients aged ≥ 75 years in the RMM cohort compared to NDMM cohort. A higher proportion of patients received bortezomib subcutaneously compared to intravenously, reflecting change in practice. A cumulative bortezomib dose of 50mg was used as cut-off for analysis because 3–4 doses of bortezomib are usually administered per cycle, at 1.3mg/m^2^ for up to 6 cycles. Typically, for a patient with a BSA of up to 2m^2^, cumulative dosage can total up to 50mg.

**Table 1:** Patient and treatment characteristics.

	Total cohort N = 272	NDMM Cohort N = 120	RMM Cohort N = 152
Age: • Median (range) • < 75 years • ≥ 75 years	69 (32–95) 175 (64%) 97 (36%)	68 (37–95) 85 (70.8%) 35 (29.2%)	71 (32–93) 90 (59.2%) 62 (40.8%)
Sex: • Male • Female	162 (60%) 110 (40%)	70 (58.3%) 50 (41.7%)	92 (60.5%) 60 (39.5%)
ASCT^1^: • Yes • No	68 (25%) 204 (75%)	31 (25.8%) 89 (74.2%)	37 (24.3%) 115 (75.7%)
Regimen: • Doublet • Triplet	114 (42%) 158 (58%)	43 (35.8%) 77 (64.2%)	71 (46.7%) 81 (53.3%)
Median number of cycles (range): • Doublet^2^ • Triplet^3^	5 (1–12) 6 (1–36)	5 (1–9) 6 (1–20)	5 (1–12) 6 (1–36)
Cumulative dose^4^^,^^5^: • <50mg • ≥50mg	148 (55%) 124 (45%)	70 (58.3%) 50 (41.7%)	78 (51.3%) 74 (48.7%)
Route of administration: • Subcutaneous • Intravenous	189 (70%) 83 (30%)	92 (76.7%) 28 (23.3%)	97 (63.8%) 55 (36.2%)
Median number of cycles (range): • Subcutaneous • Intravenous	6 (1–36) 5 (1–10)	6 (1–20) 5 (1–9)	6 (1–36) 5 (1–10)
Median follow up in months (range)	30 (1–82)	27 (1–79)	36 (1–82)

^1^ ASCT: autologous stem cell transplant

^2^doublet: bortezomib plus dexamethasone (VD)

^3^triplet: bortezomib in combination with a a corticosteroid and an alkylating or an IMiD or novel agent (see Table 2) for more details.

^4^Cumulative dose for total patients who received a transplant was as follows: ≥50mg (48.5% in total cohort, 41.9% in NDMM, 54% in RMM), <50mg (51.5% in total cohort, 58% in NDMM, 45.9% in RMM).

^5^Cumulative dose for total patients who did not receive a transplant was as follows: ≥50mg (44.6% in total cohort, 41.6% in NDMM, 46.9% in RMM), <50mg (55.4% in total cohort, 58.4% in NDMM, 53.1% in RMM).

Doublet regimen (n = 114) was a combination of bortezomib and dexamethasone (VD). The majority of patients receiving a triplet regimen had VCD (n = 123) or VTD (n = 24), or other less frequently used triplet combinations, which are all summarised in Table 2. Of the triplet cohort, 32/158 patients started on a doublet and were switched to a triplet (after one or two cycles) and 21 /158 received treatment with more than one bortezomib based triplet regimen. For patients who could not tolerate twice weekly bortezomib, weekly dosing was used to maintain as a reduced frequency schedule.

**Table 2:** Regimens used and median number of cycles.

	Number of patients			Median number of cycles (range)		
	Total (272)	NDMM (152)	RMM (120)	Total (272)	NDMM (152)	RMM (120)
VD^1^	114	43	71	5 (1–12)	5 (1–9)	5 (1–12)
VCD^2^	123	63	60	6 (1–20)	6 (1–20)	6 (1–13)
VTD^3^	21	13	8	4 (1–14)	4 (2–6)	4 (1–14)
VRD^4^	6	3	3	5 (1–36)	4 (2–5)	7 (1–36)
PanBorDex^5^	4	0	4	3 (1–5)	-	3 (1–5)
BVD^6^	8	4	4	4 (1–8)	4 (1–5)	6 (3–8)
VMP^7^	3	0	3	5 (2–7)	-	5 (2–7)
BorDoxDex^8^	3	3	0	4 (3–6)	4 (3–6)	-
Tabalumab-BorDex^9^	2	1	1	6 (3–8)	8 (8)	3 (3)

VD^1^: bortezomib and dexamethasone, VCD^2^: bortezomib, cyclophosphamide and dexamethasone, VTD^3^: bortezomib, thalidomide and dexamethasone, VRD^4^: bortezomib, lenalidomide and dexamethasone, PanBorDex^5^: bortezomib, panobinostat and dexamethasone, BVD^6^: bortezomib, bendamustine and dexamethasone, VMP^7^: bortezomib, melphalan and prednisolone, BorDoxDex^8^: bortezomib, doxorubicin and dexamethasone, Tabalumab-BorDex^9^: bortezomib, tabalumab and dexamethasone

**Fig 1:**
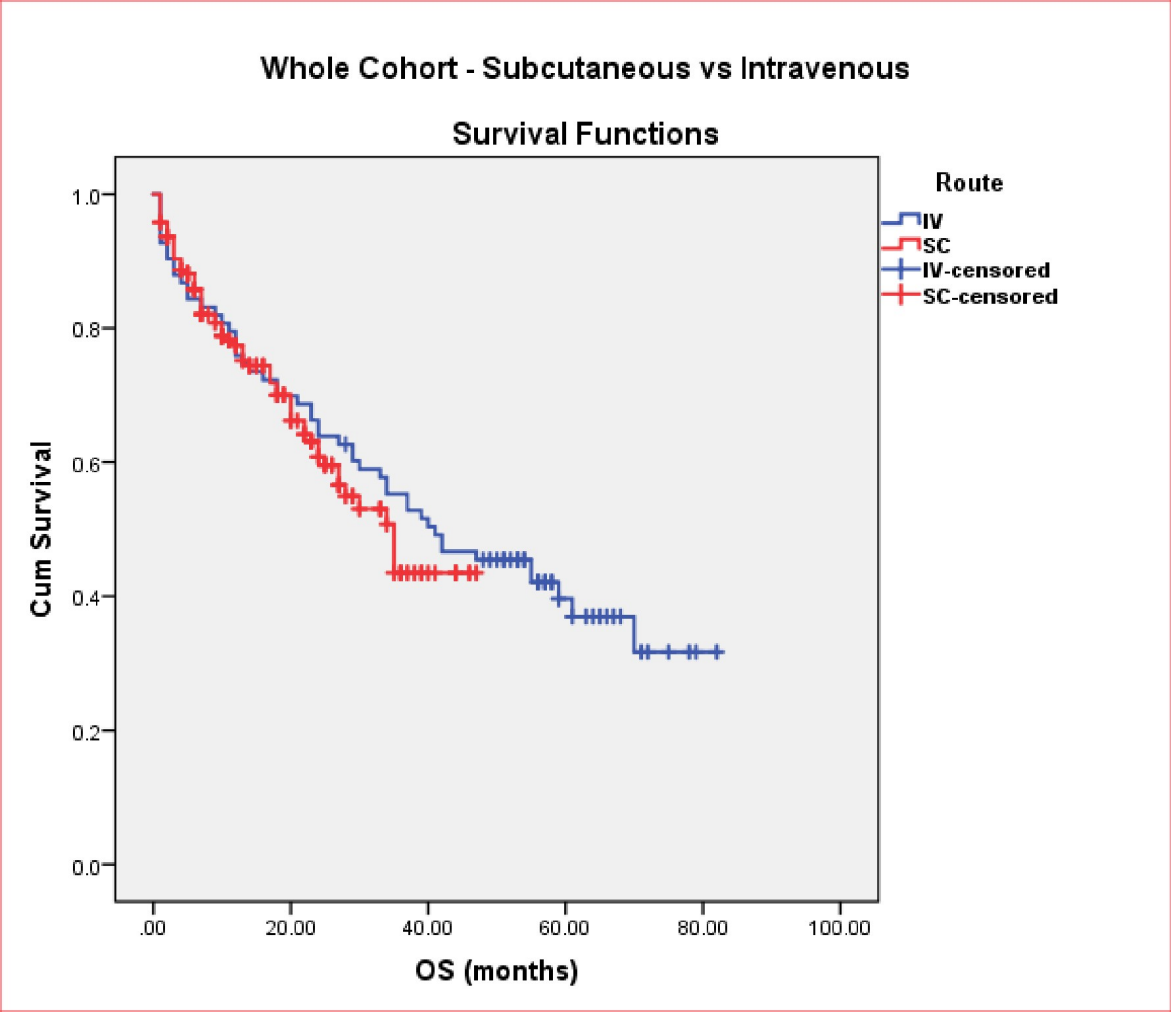
OS in total cohort (sc vs. iv).

Bortezomib route of administration transitioned from iv to sc in 2014. We audited 189 patients treated iv versus 89 treated with sc for either NDMM or RMM. Median cycle numbers were iv: 5 (1–10) and sc: 6 (1–36), respectively. Route of administration (iv vs. sc) did not influence OS (41 vs 35 months, p = 0.5) (Fig 1), or TTNT (14 vs. 19 months, p = 0.052) outcomes. Dose intensity analysis demonstrated a statistically significant improvement in median OS in patients receiving a cumulative dose of ≥50mg compared to <50mg (42 vs. 33months, p = 0.003). We have undertaken an in-depth analysis to identify reasons in the 148 patients who received a cumulative dose < 50mg. In 65/148 patients (44%), physicians recorded achievement of treatment goal as the reason for discontinuing therapy. Other causes for low cumulative dose include therapy change due to intolerable toxicities (in 22/148 patients = 15%), and documented disease progression according to IMWG criteria (in 18/148 patients = 12%). The latter was managed by, either switching to lenalidomide or palliation. A small proportion of patients 24/148 (= 16%) patients died before a cumulative dose of 50mg was delivered (10 and 14 in NDMM and RMM settings, respectively). Comorbidities, disease progression (but not proven on bloods results or imaging) were the principal reasons for early mortality. Reason for low dose was not documented in 10/148 (= 7%). Results of univariate analysis for OS in combined cohort are presented in Table 3. Univariate analysis shows that age (<75 years), transplant (i.e. for patients who received ASCT), and cumulative dose (≥50mg) are all statistically significant factors associated with improved OS (p<0.05). Combination therapy (i.e. use of triplet over doublets) was not statistically significant (p = 0.022).

**Table 3:** Univariate cox regression analysis for total cohort.

Variable	P value	Hazard Ratio (HR)	95% CI for HR
Sex	0.548	1.12	0.751–1.488
Route	0.536	0.881	0.479–1.283
Age	0.0001	0.486	0.115–0.856
combination	0.022	1.549	1.175–1.923
Transplant	0.0001	3.169	2.591–3.747
Cumulative dose	0.004	1.737	1.363–2.111

**Fig 2:**
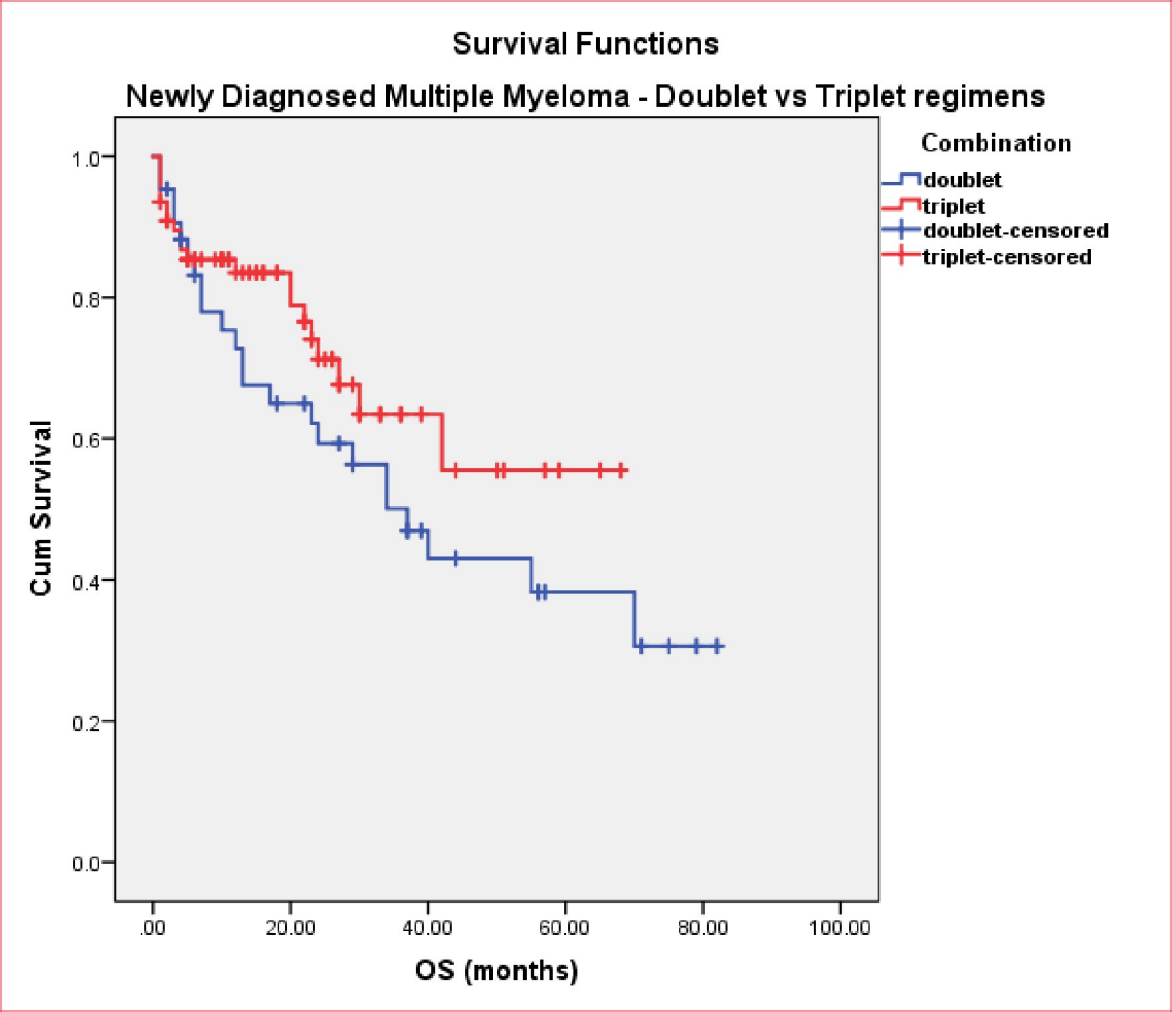
OS in NDMM (doublet vs. triplet).

**Fig 3:**
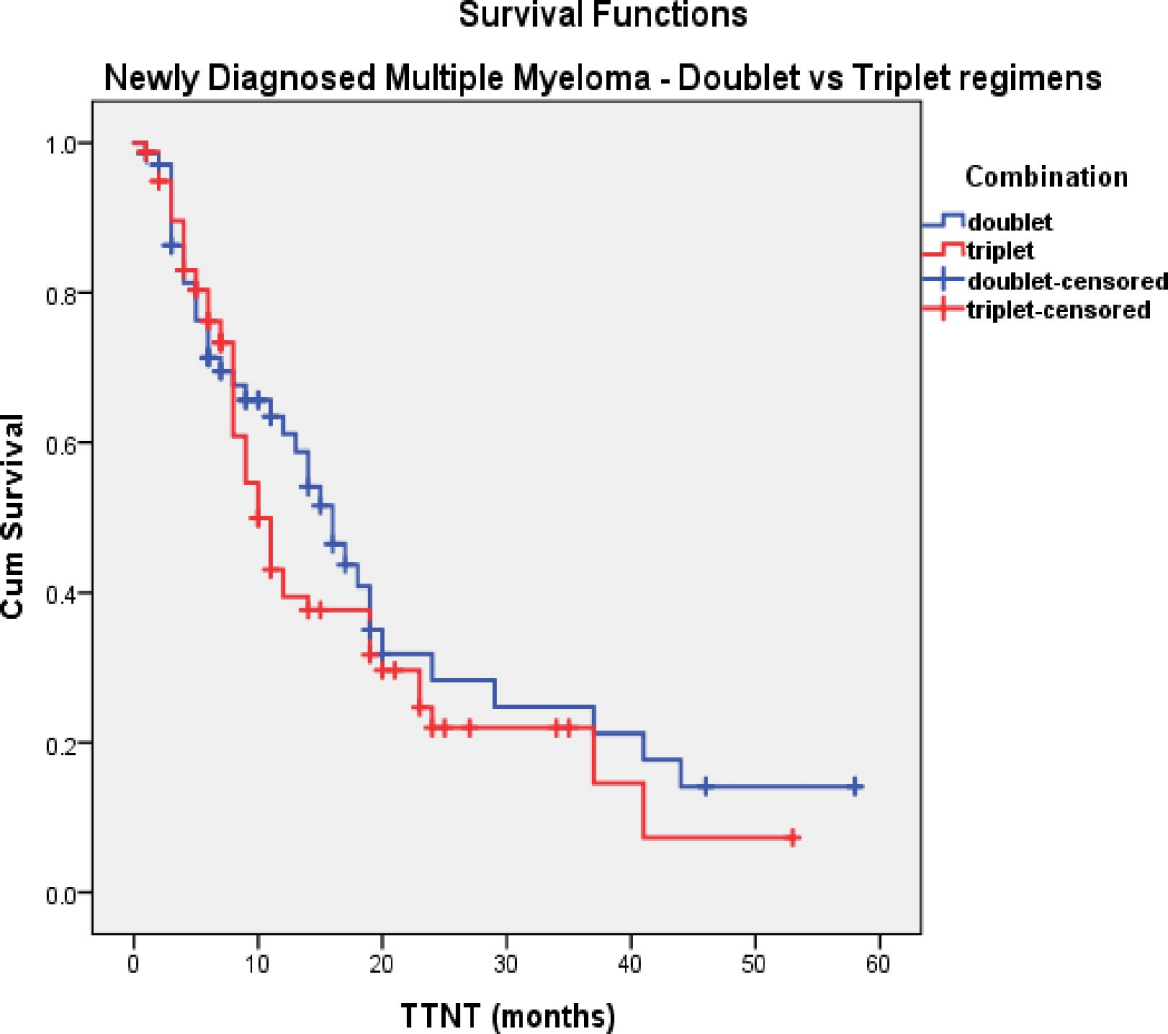
TTNT in NDMM (doublet vs. triplet).

**Fig 4:**
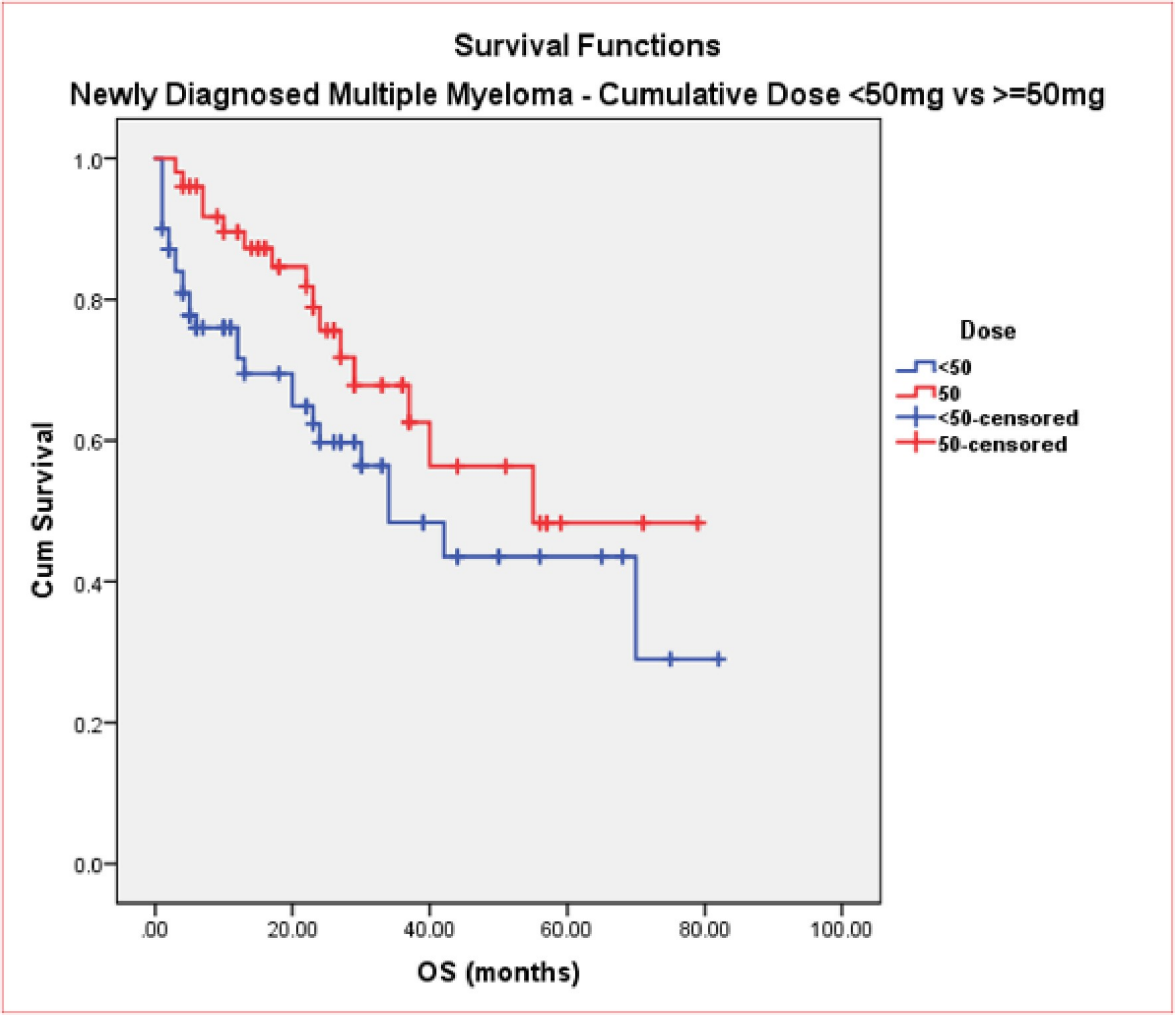
OS in NDMM (≥50mg vs. <50mg).

In the NDMM cohort, sex disposition did not make a significant difference to OS (40months vs. not reached, p = 0.4). As expected, patients who received an ASCT have an improved OS compared to TNE patients (not reached vs. 34months, p = 0.001). Patients on either doublet or triplet bortezomib regimens received similar numbers of cycles (median 5 vs. 6, respectively). Median OS was in favour of using triplet therapies compared to doublets treatment, but not reaching statistical significance (not reached vs. 37months, p = 0.19) (Fig 2). Median TTNT, although longer in patients receiving triplet therapy, was not statistically significant (34 vs. 23 months, p = 0.8) (Fig 3). Cumulative dose of bortezomib ≥50mg resulted in a longer median OS (55 vs. 34 months, p = 0.07) (Fig 4) but without reaching a statistical significance. Results of univariate analysis for OS in NDMM cohort are presented in Table 4. Univariate analysis shows that age, transplant are significant factors associated with improved OS (p<0.05)

**Table 4:** Univariate cox regression analysis for NDMM cohort.

Variable	P value	Hazard Ratio (HR)	95% CI for HR
Sex	0.418	0.771	0.142–1.4
Route	0.528	0.797	0.091–1.503
Age	0.041	0.525	-0.092–1.142
Combination	0.201	1.494	0.878–2.109
Transplant	0.004	7.848	6.427–9.269
Cumulative dose	0.079	1.756	1.127–2.385

**Fig 5:**
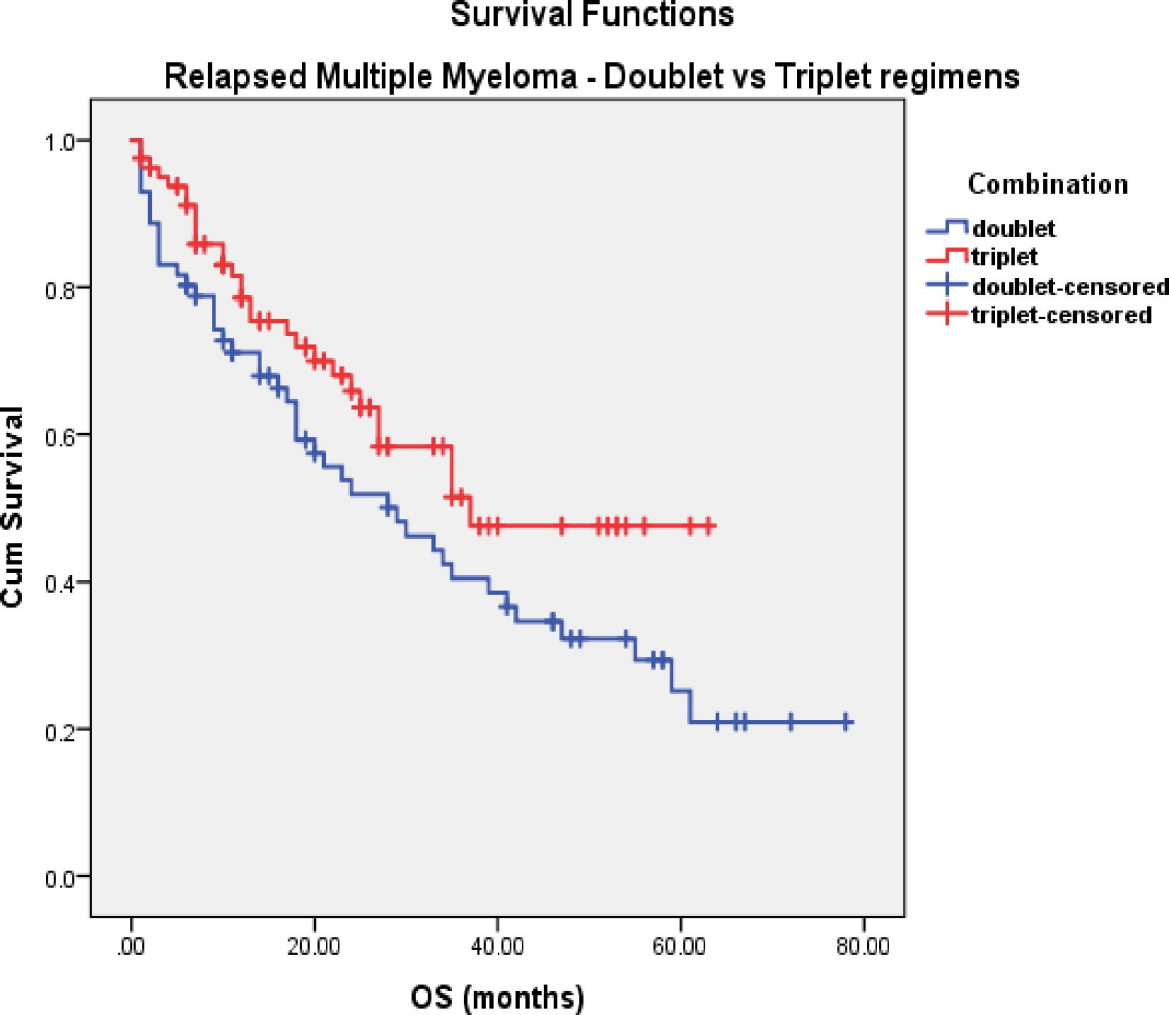
OS in RMM (doublet vs. triplet).

**Fig 6:**
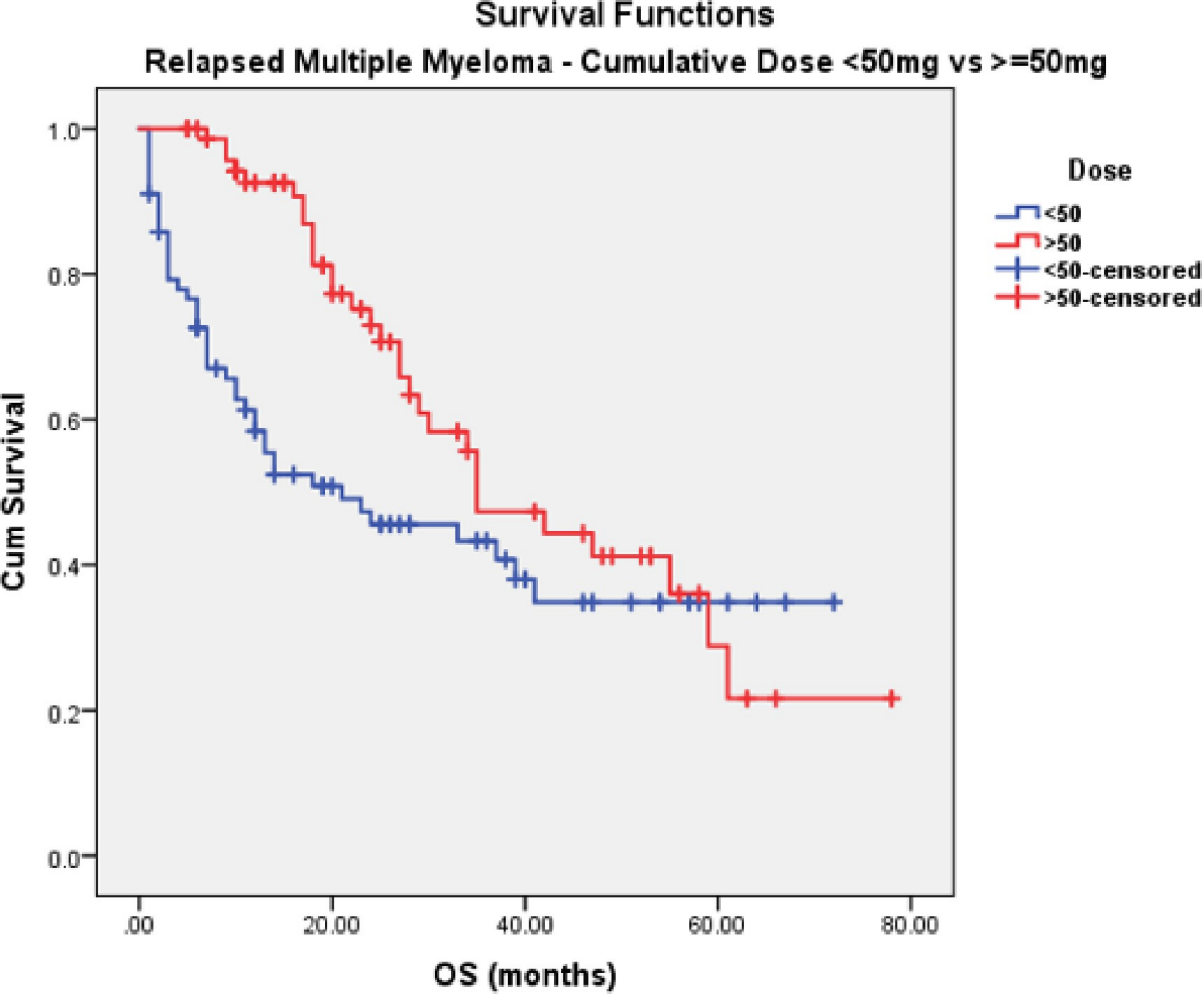
OS in RMM (≥50mg vs. <50mg).

In the RMM cohort, patients on doublet and triplet bortezomib regimens received similar numbers of cycles (median 5 vs. 6, respectively). Median TTNT was longer following treatment with a doublet regimen, but the difference was not statistically significant (16vs. 10 months, p = 0.36). Paradoxically, OS was longer when triplet therapy was used compared to doublets but not reaching statistical significance (37 vs. 29 months, p = 0.06) (Fig 5). A cumulative bortezomib dosage of ≥50mg resulted in longer OS but the difference remained statistically insignificant (35 vs. 21 months, p = 0.07) (Fig 6). Results of univariate analysis for OS in RMM cohort are presented in Table 5.

**Table 5:** Univariate cox regression analysis for RMM cohort.

Variable	P value	Hazard Ratio (HR)	95% CI for HR
Sex	0.152	1.4	0.939–1.861
Route	0.616	0.881	0.387–1.375
Age	0.002	0.468	0–0.938
Combination	0.074	1.54	1.066–2.014
Transplant	0.01	2.324	1.681–2.967
Cumulative dose	0.016	1.778	1.311–2.244

To investigate factors influencing OS outcomes, a multivariate Cox Regression analysis was performed and is presented in Table 6. A cumulative bortezomib dose ≥50mg (p = 0.002, HR = 1.83, 95% CI 1.25–2.67) and ASCT (p = 0.002, HR = 2.6, 95% CI 1.41–3.98) were both significant factors associated with improved OS, independent of sex, combination and age. A further analysis was conducted to investigate the effect of age on OS. A higher cumulative dose (≥50mg) extended OS significantly in patients aged <75 years, compared to <50mg (55 vs. 42months, p = 0.03). In older patients (> 75 years), however, median OS periods were considerably shorter but cumulative dose of ≥50mg group had a better outcome (29 vs. 18months, p = 0.06).

**Table 6:** Multivariate cox regression analysis.

Variable	P value	Hazard Ratio (HR)	95% CI for HR
Sex	0.718	0.934	0.643–1.356
Treatment line	0.148	0.756	0.517–1.104
Age	0.075	0.697	0.468–1.037
Combination	0.512	1.141	0.769–1.694
Transplant	0.002	2.651	1.412–4.979
Cumulative dose	0.002	1.827	1.248–2.675

## Discussion

The use of bortezomib in clinical practice is considerably different in comparison with the published regimens in Phase III trials APEX and VISTA [11, 12]. Understanding clinical outcomes of bortezomib usage in clinical practice would provide information for us to optimise use of this effective anti-myeloma therapy. Evidence of similar efficacy of subcutaneous compared to intravenous route of bortezomib administration, both at diagnosis, relapse and in frail patients has been published [13]. A large Czech retrospective study (n = 446) reported similar response rates of sc vs. iv routes both at NDMM and RMM settings regardless of transplant status, but with no reduction in incidence and grading of peripheral neuropathy [14]. A recent large Italian retrospective study (n = 358) established an ORR of 90% using sc compared to 80% using iv route (p = 0.118) in 326 TE NDMM treated with either VTD or VD [13]. Our data adds to the body of evidence reporting no difference in survival based on route of drug administration, although we did not examine response rates, or toxicity outcomes in these cohorts.

Our study found that a cumulative bortezomib dose ≥50mg is associated with longer survival and delayed relapse. However, due to the retrospective nature of study design, and a proportion of early deaths leading to reduction in cumulative dose, interpretation of the effect of bortezomib dose is limited by confounders. Nevertheless, it can be argued that these patients could benefit from staying on prescribed duration of bortezomib therapy in order to deepen and/or extend myeloma response, and consequently improve OS. One of the established reasons for a low cumulative dose was physicians’ strategy to manage toxicities by prescribing lower than recommended doses despite extending beyond a duration of 5 cycles. Our study found that more patients received a cumulative dose <50mg compared to those who received ≤5 cycles of treatment (148 vs. 121), which is reflective of physician’s schema of active dose reductions to manage toxicity and improve tolerability. In these patients, continued bortezomib therapy to deliver a high cumulative target dose, will improve clinical outcomes.

Mateos et al previously investigated effect of cumulative dose of bortezomib in VISTA trial where median cumulative dose in 340 patients who received VMP was 39mg/m^2^ [15]. OS was significantly longer in the higher cumulative dose group (median 66.3 (≥39mg /m^2^) vs. 46.2 months (<39mg/m^2^), p < 0.0001) [15].

In this study, we also investigated the impact of choice of doublet versus triplet bortezomib regimen on OS and TTNT in NDMM and RMM patients. Although, triplet therapy was associated with longer OS compared to doublets in both myeloma setting, difference did not reach statistical significance (NDMM P = 0.19, RMM p = 0.06). Impact of triplet vs. doublet on TTNT in either myeloma settings was shown to be modest. According to both univariate and multivariate analyses, the combination of bortezomib therapy had limited effect according to our data. Any effect observed due to combination is confounded by the transplant and cumulative dose received by patients.

Our data supports the findings of Upfront study, a community-based large phase 3 trial of NDMM, which showed no difference in median PFS between VD (doublet), VTD (triplet) and VMP (triplet), (14.7 vs. 15.4 vs. 17.3 months, respectively) [16]. All of VD, VTD and VMP treatment arms were characterised by high numbers of elderly patients ≥75 (50% vs. 38%, and 37%, respectively), and this is consistent with our cohort of patients [16]. Mean bortezomib dose intensities for VD, VTD, and VMP during induction were 72%, 63%, and 68%, respectively. During maintenance, dose intensities were 75%, 81%, and 87%, respectively [16]. Maintenance bortezomib was received by 40% of patients (<50%) [16]. Our findings suggest that in routine clinical practice, a significant proportion of patients are unlikely to reach a cumulative bortezomib dose ≥50mg, and the reasons are multifactorial. Physicians should aim to achieve a deeper myeloma response using high cumulative doses as this could increase proportion reaching a minimal residual disease (MRD) negative state, which is increasingly associated with improved PFS/OS. There is a growing understanding within the myeloma community that a fair number of patients are frail and unfit at diagnosis. Palumbo et al recently led development of a frailty score, which predicts mortality and risk of treatment toxicities in myeloma patients [17]. In practice, this will help physicians to identify frail patients early, and aid their decision making in order to reduce toxicities, and improve outcomes [17]. Ongoing trials are also investigating attenuated dosing regimens in myeloma such VMP lite and VCD lite. In the UK, myeloma XIV trial will adopt a frailty index-adjusted strategy to randomise individual patients to appropriate treatment arms that fit their frailty indices [18].

## Conclusion

This large retrospective audit provides the first long–term efficacy outcomes of bortezomib usage in clinical practice in the UK, and demonstrated that a high cumulative dose (≥50mg) statistically improved overall survival. Delivering a higher cumulative dose, however, needs to be balanced with good tolerability. This study demonstrated that there can be an OS benefit from use of bortezomib based triplets over doublets, although the impact on TTNT was modest.
